# Quality of life of 26 family members from four generations with X-linked hypophosphatemia: a cross-sectional study

**DOI:** 10.3389/fendo.2025.1544016

**Published:** 2025-04-14

**Authors:** Afaf I. Alsagheir, Bassam Bin-Abbas, Nujud M. Alghamdi, Raghad T. Alhuthil, Sarah A. Murad, Tala H. Husein, M. Zulf Mughal, Zehour E. Alsabban, Layla M. Almarzoug, Khushnooda Ramzan

**Affiliations:** ^1^ Department of Pediatrics, King Faisal Specialist Hospital & Research Centre, Riyadh, Saudi Arabia; ^2^ College of Medicine, Alfaisal University, Riyadh, Saudi Arabia; ^3^ Department of Nursing Development and Saudization, King Faisal Specialist Hospital & Research Centre, Riyadh, Saudi Arabia; ^4^ Department of Radiology, King Faisal Specialist Hospital & Research Centre, Jeddah, Saudi Arabia; ^5^ Department of Pediatric Endocrinology, Al Jalila Children’s Specialty Hospital, Dubai, United Arab Emirates; ^6^ College of Medicine, Princess Nourah bint Abdulrahman University, Riyadh, Saudi Arabia; ^7^ Department of Clinical Genomics, King Faisal Specialist Hospital & Research Centre, Riyadh, Saudi Arabia

**Keywords:** X-linked hypophosphatemia, quality of life, complications, pain, physical function

## Abstract

**Introduction:**

X-linked hypophosphatemia (XLH) is a lifelong, progressive genetic condition affecting patients’ physical health and quality of life.

**Methods:**

This cross-sectional study aimed to understand the burden of XLH on four generations of family members with XLH. 26 family members with XLH from Saudi Arabia were assessed via a home visit and clinical assessment in hospital. Patient demographics, biochemical parameters, and radiological and skeletal ndings were collected. Quality of life was assessed using the 36-Item Short Form Survey (SF-36) and Pediatric Quality of Life Inventory (PedsQL 4.0). Further assessment involved the 6-minute walk test (6MWT) and Western Ontario and McMaster Universities Arthritis Index (WOMAC) pain assessment.

**Results:**

Our results showed low quality of life for the adults and children, with mean SF-36 and PedsQL (8–18 years) scores of 34.12 (standard deviation [SD] 25.02) and 55.04 (SD 29.47), respectively. High levels of complications of XLH and surgical interventions were common, including dental abscesses (92%), tooth loss (73.07%), osteotomies (76.92%) and craniosynostosis (76.90%). In 15 adult patients, aged 35–55 years, moderate WOMAC scores for pain, stiffness, and function of hip and knee joints and low 6MWT scores were reported. Skeletal deformities in the hip (53.85%) and skull (76.90%), and fractures and pseudofractures (38.40%), were common among older patients.

**Discussion:**

These ndings demonstrate that the burden of XLH in these family members who had delayed diagnosis and were non-compliant to medical treatment and supportive care was high. Greater awareness and early diagnosis are essential for identi cation of cases and early initiation of treatment.

## 1 Introduction

X-linked hypophosphatemia (XLH) is a hereditary condition caused by mutations in the phosphate-regulating endopeptidase homolog X-linked (PHEX) gene (1). The loss of function of PHEX causes increased levels of fibroblast growth factor 23 (FGF23), leading to renal phosphate wasting as well as reduced serum 1,25-dihydroxyvitamin D levels, which lead to decreased absorption of phosphate and calcium from the intestines (2). Chronically reduced serum phosphate leads to impaired mineralization of the growth plate and osteoid, which in turn result in skeletal deformities, dental issues, and other systemic disorders (3). The incidence of the disease is approximately 1 in 20,000 to 1 in 70,000, according to the International XLH Registry (4).

In addition to the physical impacts of the disease, health-related quality of life is also deeply impacted in patients with XLH (1). Several studies have shown that XLH significantly impacts patients’ quality of life. Studies utilizing standardized questionnaires like the 36-Item Short Form Survey (SF-36) and EuroQol-5D have revealed that patients with XLH experience substantial limitations in physical and mental health domains, including pain, mobility, and daily activities (5, 6). These findings underscore the urgent need for targeted interventions to address the multifaceted challenges faced by individuals with XLH. Using the Oral Health Impact Profile questionnaire, Hanisch et al. examined the impact of XLH on oral health-related quality of life (HRQoL) (7). The study demonstrated that the majority of patients with XLH suffered from oral symptoms, including dental abscesses and fistulas, which negatively impacted oral HRQoL. The patients with XLH from the study had worse oral HRQoL, with a score of 10.30, compared with the general population, with a score of 4.09 (7).

Additionally, quality-of-life evaluations in XLH have gone beyond the evaluation of oral and physical health. The psychosocial effects of XLH have been investigated by Lo et al., who reported that patients experienced low self-esteem and well-being, depression, and frustration resulting from negative social encounters such as bullying (8). Furthermore, Yanes et al. reported that 65% of adult patients with XLH in their study experienced varying degrees of anxiety or depression (9). A Clinical Practice Research Datalink study in the UK demonstrated that patients with XLH were three times more likely to have depression versus a matched population of patients without XLH (10).

Conventional treatment for patients with XLH involves supplementation with phosphate and active vitamin D (5). Treatment in children aims to correct skeletal and radiographic abnormalities and lessen the effects of rickets and osteomalacia (5). In adults, treatment can help to minimize pain, improve healing of fractures or following surgery, and lessen the effects of osteomalacia. However, treatment is often discontinued once the bones are matured (5, 6). Despite treatment with conventional therapy, some patients continue to experience symptoms as well as side effects such as hyperparathyroidism, diarrhea, nephrocalcinosis, nausea, and abdominal pain (11). Dose adjustments and regular monitoring are recommended to overcome these limitations (5). Burosumab, an anti-FGF23 monoclonal antibody that increases levels of serum phosphate, has been shown to improve the symptoms of rickets and improve growth rates in children (12). In adults, burosumab therapy has been shown to lead to improved healing of fractures or pseudofractures and improvements in physical function, pain, and stiffness (13). Early diagnosis and treatment initiation is essential to prevent complications in patients with XLH. Such complications can include osteoarthritis, deafness, enthesopathies, or spinal stenosis (14).

This aims of this observational cohort study were:

- To describe patient background and demographics and to assess the quality of life in four generations of patients with XLH from the same family residing in a semi-remote area of Mecca, Saudi Arabia
- To assess the impact of XLH on joint stiffness and mobility across the life course
- To describe the current biochemical parameters and radiological and skeletal findings in these patients.

## 2 Materials and methods

This cross-sectional study was conducted at King Faisal Specialist Hospital & Research Centre (KFSHRC), Riyadh, Saudi Arabia. Following the establishment of an FGF23-related hypophosphatemic rickets and osteomalacia registry at KFSHRC and loss of follow-up of 12 family members who previously attended the hospital due to poor socioeconomic status and the COVID pandemic, the medical team from KFSHRC visited the entire family residing in a semi-remote area of Mecca, Saudi Arabia to conduct a clinical assessment. The visit was conducted on 25 June 2022 by a team of three pediatric endocrine consultants and two nurse practitioners.

Ethics approval for this study was granted by the institutional review board at KFSHRC (ethics approval number: 2211051).

### 2.1 Patient cohort

**Figure 1:**
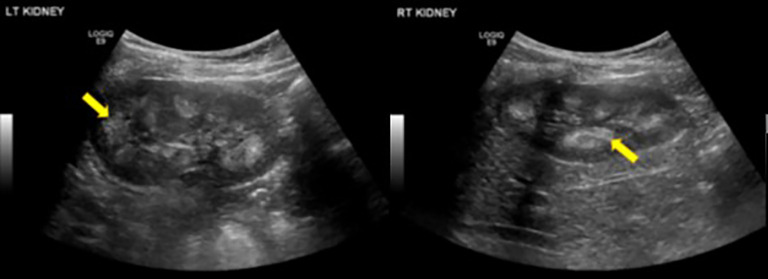
Renal Ultrasound of bilateral kidneys in a 9-year-old patient showed diffusely echogenic pyramids (yellow arrows), suggestive of bilateral medullary nephrocalcinosis.

The patient cohort consisted of 30 individuals across four generations of the same family who manifested with XLH (**Figure 1**). Of these family members, 26 were assessed during the medical teams’ visit and had genetic confirmation of pathogenic mutations in the PHEX gene. The remaining four family members did not undergo assessment or genetic testing but had skeletal manifestations and had previously been diagnosed with hypophosphatemic rickets at KFSHRC based on clinical and biochemical data. Three of these four family members were deceased at the time of study. Clinical data was available for the final family member, who was not assessed at the medical teams’ visit.

### 2.2 Clinical evaluation

During the medical teams’ visit, patients were interviewed. Information was collected using a standardized case report form regarding symptoms related to their condition (pain, muscle weakness, ability to walk and do their daily activity, fractures, skeletal deformity), medical treatment, compliance to treatment, and surgical history. Visits to the physiotherapist at KFSHRC were arranged for further clinical assessment, which included the 6-minute walk test (6MWT) and Western Ontario and McMaster Universities Arthritis Index (WOMAC) pain assessment. Growth status was assessed by comparing the latest documented weight and height or length measurements to age–gender standard deviation (SD) scores. Laboratory tests were performed in the laboratory at KFSHRC, following the clinical team visit to the family, which included fasting levels of serum calcium, serum phosphate, parathyroid hormone (PTH), serum alkaline phosphatase (ALP), tubular reabsorption of phosphate, ratio of tubular maximum reabsorption rate of phosphate to glomerular filtration rate (TmP/GFR), spot urine, creatinine, calcium, and phosphate (from second urine sample passed in the morning).

Radiological assessments included X-rays of the left hand and knee, renal ultrasound to evaluate for nephrocalcinosis, and brain and spine magnetic resonance imaging (MRI). Bone densitometry was assessed by DEXA scan for adult patients only using the GE Lunar iDXA (GE Healthcare Lunar, Madison, WI), scanning both proximal femurs and lumbar spine.

### 2.3 Genetic analysis

The proband, who continues to be followed up at KFSHRC, was a 48-year-old male. He presented with abnormal skeletal morphology, elevated circulating ALP levels, hypophosphatemia, and short stature. He was genetically screened for the PHEX gene due to these clinical manifestations. The pathogenic PHEX variant identified in the proband was c.2239C>Tp.(Arg747*) using CentoXome^®^ Solo at the CENTOGENE laboratory. Targeted screening testing for the same variant was undertaken in the rest of the family members at the CENTOGENE laboratory and supported by Kyowa Kirin Pharma.

### 2.4 Quality-of-life surveys

Family members completed validated quality-of-life surveys during the medical teams’ visit to Mecca. Two main validated Arabic-translated instruments were administered in this study to assess the quality of life of the patients with XLH: the SF-36 and the Pediatric Quality of Life Inventory (PedsQL 4.0).

The SF-36 questionnaire includes multiple-item subscales that assesses physical function, social functioning, role constraints brought on by physical difficulties, role restrictions brought on by emotional problems, mental health, vitality, pain, and overall perception of health (15). The questionnaire was self-administrated for adult patients (age >18 years). Each SF-36 subscale has a total score between 0 and 100. A higher score indicates better HRQoL.

The PedsQL 4.0 questionnaire evaluates the generic HRQoL in children and adolescents. It comprises 23 items that measure physical, emotional, social, and school functioning and has been demonstrated to be psychometrically valid and reliable. Developmentally appropriate versions were used for children and adolescents: child self-report for ages 8–12 years and 13–18 years, and interview-based parent-proxy report for children ages 5–7 years (administered by a nurse practitioner). Higher scores indicate a greater HRQoL. The score is not given if more than 50% of items are missing from a scale (16, 17).

Both questionnaires have been linguistically validated in the literature (English to Arabic) (18, 19). Nevertheless, internal consistency was measured using the Cronbach’s alpha reliability test.

### 2.5 Statistical analysis

Data analysis was performed using STATA (version 17). Continuous data were presented as mean, SD, range, median, or interquartile range, as appropriate. Categorical data were presented as frequencies and percentages.

## 3 Results

### 3.1 Patient demographics

Demographic information of the family members is summarized in **Table 1**. All adult patients (n = 19) were reported to have presented with genu varum (bowed legs) since early childhood, but only 12 patients (3 females and 9 males) sought medical advice at KFSHRC. The proband first visited KFSHRC in 1982 at the age of 8 years. The remaining 18 patients had a mean age at diagnosis of 7 years (SD 4.5) and were diagnosed based on clinical and radiological signs of rickets, as well as low mean serum phosphate (0.55 mmol/L, SD 0.32; reference range adults 0.80–1.50, children 0.90–1.80), high mean ALP (565 IU/L, SD 115.90; reference range adults 30–130, children [3–10 years] 130–260, children [10–14 years] 130–340, children [14–18 years] 30–180), and low tubular reabsorption of phosphate (reference range <75%). The patients were started on calcitriol, the active form of vitamin D, and oral phosphate supplements. However, the patients were not compliant with their treatment, and they did not follow up regularly. During their teenage years, they underwent surgical correction of their lower limbs by osteotomy.

**Table 1:** Demographic information (N = 26).

Variables	
Age (years), mean (SD) [range]	31 (16.79) [5–54]
Age categories (years), n (%)	
5–7	1 (3.85)
8–18	6 (23.08)
>18	19 (73.08)
Gender, n (%)	
Male	12 (46.15)
Female	14 (53.85)
Pediatric* height SDS, mean (SD) [range]	-2.71 (1.14) [-4.36, -1.72]
Adult** height, mean (SD) [range]	
Males	150.14 (7.81) [133–158]
Females	140.25 (5.14) [130–146]
Adult** height SDS, mean (SD) [range]	-2.41 (0.94) [-4.8, -1.4]
Adult** BMI, mean (SD) [range]	29.73 (9.04) [23–49.6]
Current management, n (%)	
None	7 (26.92)
Oral phosphate and calcitriol	16 (61.54)
Burosumab	3 (11.54)

*Reported for pediatric patients aged less than 14 years.

**Adults are those aged ≥18 years (N = 20).

BMI, body mass index; SD, standard deviation; SDS, standard deviation score.

### 3.2 Clinical evaluation

**Figure 2:**
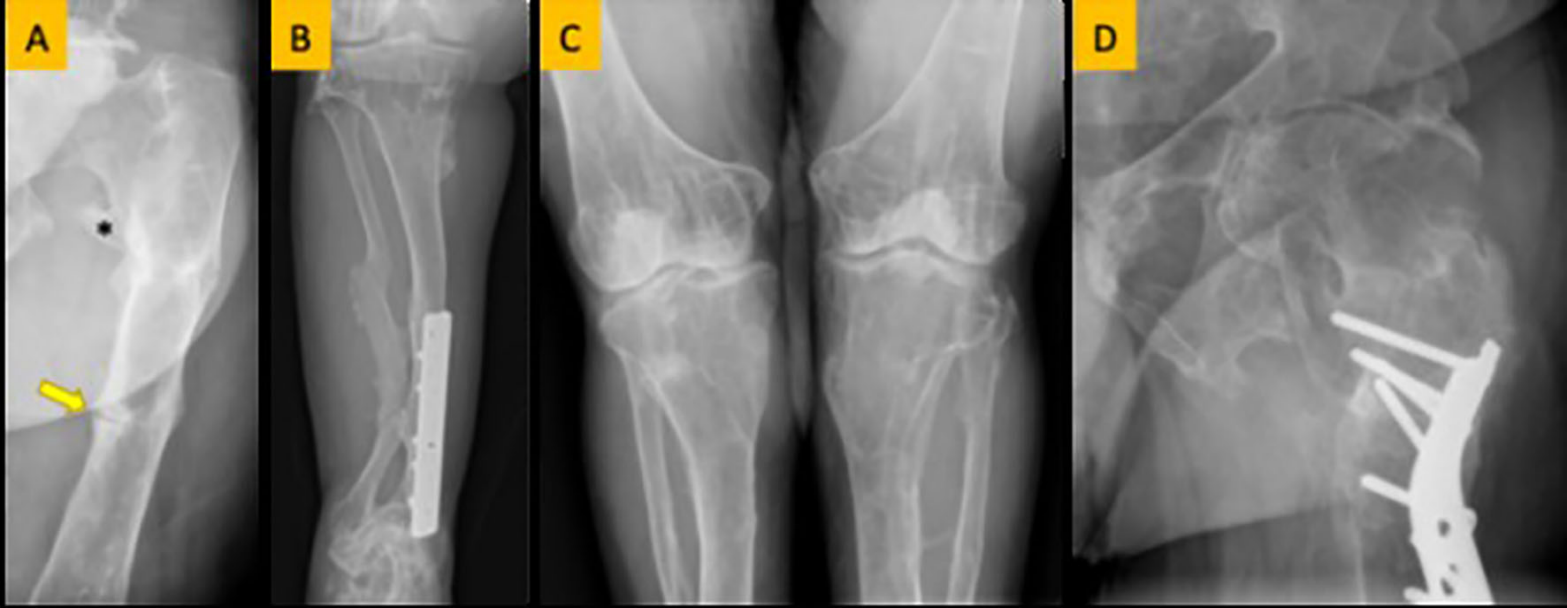
**(A)** Femoral looser zone (yellow arrow) and enthesopathy (asterisk). **(B)** Bowing deformity and remodeling of the tibia and fibula. **(C)** Genu valgus (knock knee) deformity. **(D)** Coxa valgus deformity.

**Figure 3:**
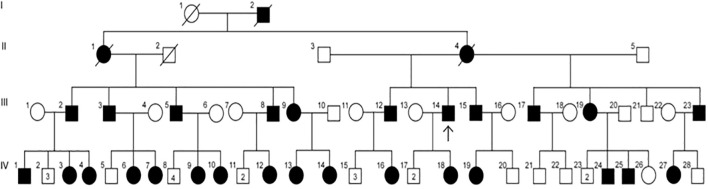
Pedigree of a family with XLH, suggesting X-linked dominant inheritance. Circles = females; squares = males; solid symbols = affected individuals; open symbols = unaffected individuals; strike-through symbols = deceased; arrow = proband; numbers within symbols = number of male or female sibling (not affected).

**Figure 4:**
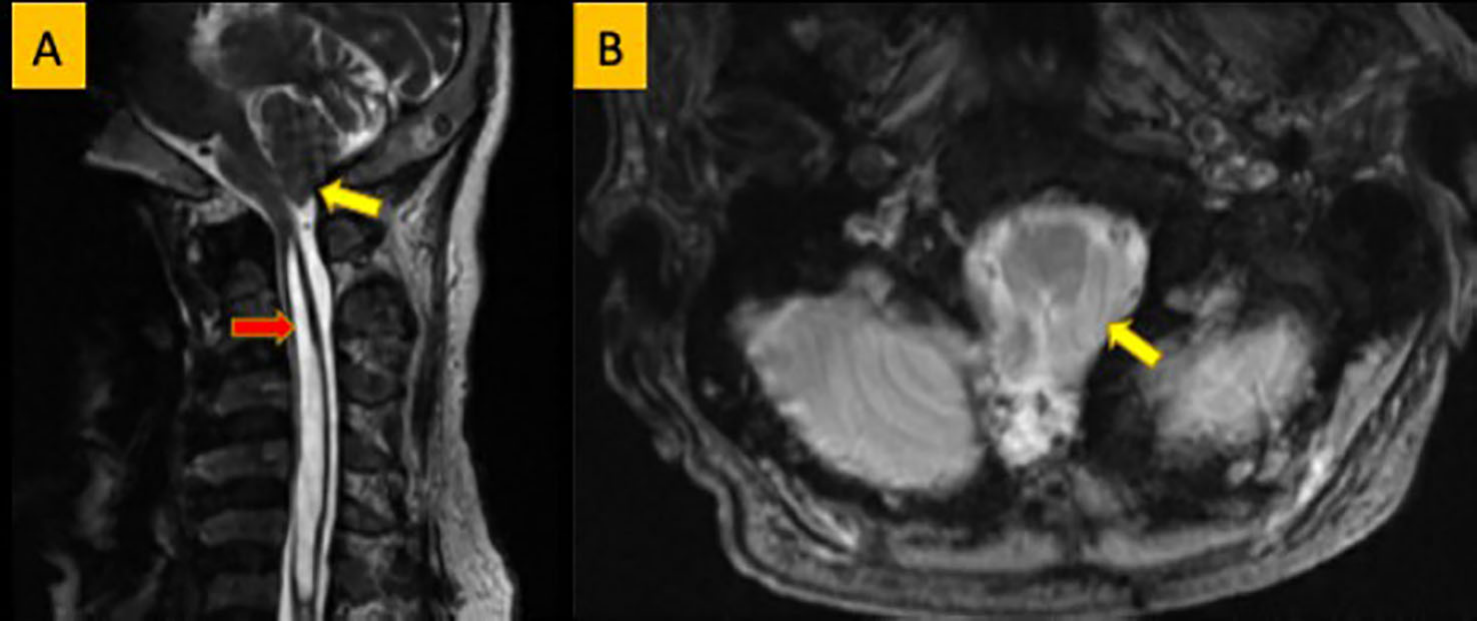
**(A)** Sagittal T2WI showing low-laying tonsil (yellow arrow). Large syrinx is seen in the cervical spinal cord (red arrow). **(B)** Axial T2WI showing crowding of the foramen magnum by the low laying tonsil (yellow arrow).

Laboratory findings revealed high mean PTH and ALP and low mean TmP/GFR in these family members (**Table 2**). Radiological and skeletal findings are shown in **Table 3**. Renal ultrasound revealed nephrocalcinosis in two patients. Common skeletal abnormalities in the family members included skull shape abnormality (N = 20), coxa vara or valga (N = 14), and genu varum or valgum (N = 11). **Figures 2**–**4** show examples of renal ultrasound, X-ray, and MRI results.

**Table 2:** Laboratory findings at presentation (n=26).

Variables	Mean (SD)	Range	Reference Range
PO_4_, mmol/L	0.76 (0.24)	0.47, 1.21	–
Children (≤16 years)	1.04 (0.17)	0.82, 1.21	0.90–1.80
Adults (>16 years)	0.66 (0.18)	0.47, 1.15	0.80–1.50
PTH, ng/L	70.57 (38.02)	24, 164.1	15–65
Ca, mmol/L	2.36 (0.10)	2.25, 2.56	2.1–2.54
ALP, IU/L	149 (87)	90, 430	–
Children 3– <10 years	510 (49.50)	475, 545	130–260
Children 10– <14 years	432 (91.92)	367, 497	130–340
Children 14–18 years	151 (7.07)	146, 156	30–180
Adults >18 years	118 (38.58)	79, 233	30–130
25(OH)D, nmol/L	38.846 (17.20)	9, 58	<50
1,25(OH)_2_D, pmol/L	140 (51.41)	82, 180	48–192
TRP, %	55.2 (9.4)	40, 68	<75
Tmp/GFR, mmol/L	0.52 (0.12)	0.32, 0.7	–
Children (≤18 years)	0.66 (0.07)	0.59, 0.74	1.15–2.44
Adults (>18 years)	0.63 (0.11)	0.45, 0.83	0.80–1.35

1,25(OH)_2_D, 1,25-dihydroxyvitamin D₃; 25(OH)D, 25-hydroxyvitamin D₃; ALP, alkaline phosphatase; Ca, serum calcium; PO_4_, serum phosphate; PTH, parathyroid hormone; SD, standard deviation; TmP/GFR, ratio of tubular maximum reabsorption rate of phosphate to glomerular filtration rate; TRP, tubular reabsorption of phosphate.

**Table 3:** Radiological and skeletal findings.

Variables	N (%)
Renal ultrasound (n = 26)	
Bilateral nephrocalcinosis	2 (7.7)
Brain MRI (n = 10)*	
Chiari malformation (required surgery)	1 (10.00)
Normal	9 (90.00)
Spine MRI (n = 10)*	
Osteoarthritis and non-expansile syrinx endplate wedging	1 (10.00)
Unremarkable	9 (90.00)
Bone density (n = 9)*	
Osteoporosis (T score ≤-2)	3 (33.33)
Skeletal findings (n = 26)	
Knee deformity: genu varum or valgum	11 (42.3)
Hip deformity: coxa vara or valga	14 (53.85)
Skull shape abnormality	20 (76.9)
Fracture/pseudofracture (looser zone)	10 (38.4)
Osteoarthritis	14 (53.8)
Enthesopathy (boney insertion sites of tendons and ligaments)	11 (42.3)
Periosteal hyperostosis (i.e. muscular attachment/interosseous membranes/syn)	6 (23.07)
Scoliosis	3 (11.53)

*All patients were adults.

MRI, magnetic resonance imaging.

Physiotherapy assessment results (**Table 4**) showed low mobility, assessed using the 6MWT, and moderate levels of pain, stiffness, and function, measured using WOMAC. Patient procedures and complications are shown in **Table 5**. Dental abscesses and tooth loss were the most common complications, as well as osteoarthritis and craniosynostosis. Of the 20 adult patients, 1 (5%) is currently bedridden and 2 (10%) are using a wheelchair.

**Table 4:** Physiotherapy assessment results.

Variables	N = 15*
6MWT, n (%)**	
Walked 200-400 m	4/15 (26.67)
Walked <200 m	6/15 (40.00)
Unable to do 6MWT using mobility aid or wheelchair	5/15 (33.33)
WOMAC (knee), mean (SD)	
Pain (0–20)	11 (4)
Stiffness (0–8)	5 (2)
Function (0–68)	28 (15)
WOMAC (hip), mean (SD)	
Pain (0–20)	9 (5)
Stiffness (0–8)	4 (3)
Function (0–68)	32 (19)

*Patient age range: 35 to 55 years; **6MWT normal range: 500-700 m.

6MWT, 6-minute walk test; SD, standard deviation; WOMAC, Western Ontario and McMaster Universities Arthritis Index.

**Table 5:** Procedure/complications.

Variables	N (%)
At least one osteotomy	20 (76.92)
Knee or hip osteoarthritis	14 (53.84)
Knee or hip replacement	0 (0.00)
Enthesopathy	14 (53.84)
Scoliosis	3 (11.53)
Dental abscesses	24 (92.00)
Tooth loss	19 (73.07)
Hearing impairment – conductive hearing loss	3 (11.38)
Parathyroidectomy	0 (0.00)
Craniosynostosis	20 (76.90)
Surgery for craniosynostosis	3 (11.54)

### 3.3 Genetic analysis

Family members were confirmed to be hemizygous for a C>T nucleotide substitution in exon 22 of the PHEX gene, resulting in the replacement of an arginine codon (CGA) with a stop codon (TGA) at amino acid position 747. This mutation is denoted as c.2239 C>T at the cDNA level or p.Arg747 top (R747X) at the protein level. According to Human Gene Mutation Database Professional release 2022.1, this variant has previously been associated with disease causing hypophosphataemic rickets by Francis et al. (20), Dixon et al. (21), and Hernandez-Frias et al. (22). In addition, well-established *in vitro* or *in vivo* functional studies are supportive of a damaging effect on the gene or gene product (23). ClinVar (National Library of Medicine) lists this variant as “pathogenic/likely pathogenic” under variation ID279873. The variant is also classified as “pathogenic” (class 1) according to the recommendations of CENTOGENE and the American College of Medical Genetics.

### 3.4 Quality of life

Results of the SF-36 and PedsQL surveys and Cronbach’s alpha reliability test are shown in **Table 6**. The SF−36 questionnaire was applied to adult participants (>18 years, n = 19) with a mean score of 34.12. The lowest scores were in the role limitations due to physical and emotional health domains (mean 23.75 and 26.67, respectively). For PedsQL (ages 8–18 years, n = 6), the overall mean score was 55.04. School functioning and emotional functioning scored the lowest (means of 48.96 and 52.5, respectively). For the PedsQL (ages 5–7 years), only one patient was tested, with an overall score of 34.8. They scored the lowest in the social functioning domain, with a score of 20. Both SF-36 and PedsQL (8–18 years) achieved excellent internal reliability scores (0.9682 and 0.9689, respectively).

**Table 6:** SF-36 and PedsQL scores and reliability test results.

Scale	Mean ± SD	Median [IQR]	Number of items (N)	Cronbach’s alpha score	Interpretation
SF-36 questionnaire (n = 19) Domain [score range 0–100]					
Physical functioning	27.25 ± 33.34	12.5 [0, 47.5]	10	0.9657	Excellent
Role limitations due to physical health	23.75 ± 37.59	0 [0, 25]	4	0.8879	Good
Role limitations due to emotional problems	26.67 ± 41.32	0 [0, 50]	3	0.8990	Good
Energy/fatigue	44.33 ± 25.69	47.5 [25, 65.8]	4	0.7480	Acceptable
Emotional well-being	50.5 ± 22.07	49 [34, 68]	5	0.7264	Acceptable
Social functioning	36.88 ± 30.21	37.5 [6.25, 50]	2	0.7348	Acceptable
Pain	31.25 ± 29.59	27.5 [0, 47.5]	2	0.8268	Good
General health	37.5 ± 22.8	32.5 [20, 45]	5	0.8155	Good
Health change	27.5 ± 26.78	25 [0, 50]	1	–	–
Overall	34.12 ± 25.02	30.94 [13.89, 41.89]	36	0.9682	Excellent
PedsQL 8-18 questionnaire (n = 6) Domain [score range]					
Physical functioning [0–32]	53.13 ± 34.69	51.56 [34.38, 87.5]	8	0.9555	Excellent
Emotional functioning [0–20]	52.5 ± 27.7	60 [50, 65]	5	0.8996	Good
Social functioning [0–20]	65.83 ± 37.07	77.5 [50, 95]	5	0.9521	Excellent
School functioning [0–20]	48.96 ± 32.81	51.87 [25, 80]	5	0.9152	Excellent
Total score [0–92]	55.04 ± 29.47	66.3 [44.32, 76.14]	23	0.9689	Excellent
PedsQL 5–7 questionnaire (n = 1) Domain [score range]					
Physical functioning [0–32]	37.5	37.5	8	–	NA
Emotional functioning [0–20]	40	40	5	–	NA
Social functioning [0–20]	20	20	5	–	NA
School functioning [0–20]	40	40	5	–	NA
Total score [0–92]	34.8	34.8	23	–	NA

IQR, interquartile range; NA, not applicable; PedsQL, Pediatric Quality of Life Inventory; SD, standard deviation; SF-36, 36-Item Short Form Survey.

## 4 Discussion

This cross-sectional study of 26 patients described the impact of XLH on four generations of patients from the same family who resided in a semi-remote area of Mecca, Saudi Arabia, and who have had delayed diagnosis and poor access to medical treatment and supportive care over the past 50 years. All family members who underwent genetic testing were confirmed to have the c.2239C>Tp.(Arg747*) PHEX variant. Quality of life was low in this set of patients, with adults reporting low scores in role limitations due to physical and emotional health domains and children having low scores in school functioning and emotional functioning domains. High levels of complications and procedures were also reported in this group of patients, including dental abscesses, tooth loss, osteotomies, craniosynostosis, enthesopathy, and hip or knee arthritis. Moderate levels of pain, stiffness, and function were recorded for hip and knee joints, and low mobility was also reported. Skeletal deformities in the knee, hip, and skull, as well as fractures and pseudofractures, were common among the patients. Biochemical testing revealed high mean PTH and ALP and low mean TmP/GFR in these family members. Raised PTH in this set of patients is potentially a result of concomitant vitamin D and/or dietary calcium deficiency, or an inadequate dose of calcitriol in comparison to oral phosphate supplements.

Low quality of life in adults with XLH, as reported using the SF-36, has been described in other studies. In a French prospective cohort study of 52 adults with XLH, mean SF-36 physical component score (PCS) was 49.5 (SD 20.5) and mean SF-36 mental component score (MCS) was 57.9 (SD 21.3) (24). This French study reported similarly high levels of complications and procedures, with 95.6% having deformities in the lower limbs, 62.5% with dental defects, 61.5% with structural lesions, 64.0% with enthesopathies, and 85.4% having radiological arthritis. Furthermore, the majority of patients were on conventional therapy, with 64.6% taking oral phosphate, 59.2% vitamin D, and 66.7% vitamin D analogs (24). A further prospective cohort study conducted in the UK reported overall mean SF-36 PCS of 32.67 (SD 14.19) and SF-36 MCS of 48.38 (10.41) for 48 adult patients with XLH (25). In the XLH Burden of Disease Survey conducted in 232 adult patients with XLH, SF-36 PCS was 37.0 and MCS was 45.9, demonstrating low quality of life in this set of patients despite the use of conventional therapy (1). These patients also reported bowing of the legs (77%), surgical procedures (94%), history of fractures and pseudofractures (40%), and dental abscesses (82%), showing similar levels of complications and procedures to our study participants (1).

Quality of life in pediatric patients with XLH, as reported using PedsQL, has also been reported in other studies. One study reported a mean PedsQL score of 75.62 (SD 0.31) in 59 pediatric patients with XLH prior to initiation of a patient support program, with emotional functioning being the lowest domain with a mean of 65.75 (SD 0.31) (26). In the same study, the PedsQL score was even lower in the subset of patients who were not receiving treatment at enrollment, with a mean of 71.28 (SD 0.32). Further studies, using different measures of HRQoL, such as the EuroQol-5D, have also shown reduction in HRQoL in adults and children with XLH (9). Quality-of-life scores were much lower in our study, potentially due to delayed diagnosis and poor access to medical treatment and supportive care experienced by the patients.

Ambulatory function (6MWT) and patient-reported joint pain, stiffness, and physical function (WOMAC) have been assessed in clinical trials of patients with XLH. In a phase 3 extension trial, adult patients with XLH had reduced ambulatory function at baseline with a mean 6MWT of 362.0 meters (SD 106.3) (27). Furthermore, according to WOMAC, patients had reduced physical function as well as stiffness and pain at baseline, with a mean of 47.4 (SD 20.0) for physical function, 63.1 (SD 20.5) for stiffness, and 49.3 (16.8) for pain (27). WOMAC was also assessed in the XLH Burden of Disease Survey, where adult patients with XLH had mean scores of 40.8 for physical function, 39.5 for pain, and 50.3 for stiffness, which were much higher than the United States normative values (1). In a prospective cohort study, Orlando et al. reported a mean 6MWT of 335 meters (SD 102) in 26 adult patients XLH, which is 38.6% less than the distance expected based on the age and gender of patients (28). This group of patients had similar levels of skeletal deformities to our study participants, including 56% with genu varum, 32% with genu valgus, and osteoarthritis of the hip or knee in 44% and 28%, respectively. Furthermore, 23% were receiving no treatment and 57.7% were being treated with conventional therapy (28). The WOMAC scores in the subset of patients from our study were lower than previous study findings mentioned above, indicating less pain and stiffness and less impaired physical function in our cohort of patients.

Patients in this study were largely untreated, resulting from poor compliance to medical treatment and supportive care, or were undiagnosed because they did not seek medical advice, which might explain the high levels of complications and procedures in these family members. Clinical complications, such as pain, stiffness, dental problems, and skeletal abnormalities, as well as the effects of these clinical symptoms on daily life, social life, mental health, and functional capabilities, are commonly reported in patients with XLH and worsened by limited access to treatment (29). In a Japanese study of 25 adult patients, ossification of spinal ligaments (80%), nephrocalcinosis (72%), hearing impairment (32%), and osteophytes in the hip and knee joints (96% and 68%, respectively) were commonly reported complications (30). In addition to these, fractures and pseudofractures, deformities of the lower extremities, and gait abnormalities are further complications reported in adults (31).

Burosumab has shown promising results in improving the quality of life of patients with XLH. A phase 3 extension study reported improvements in pain, fatigue, and ambulatory function after 48 weeks of burosumab use (32). An observational study of children at a London hospital demonstrated an improvement in PedsQL at 9 months (mean 80.9, SD 15.1) compared with baseline (mean 66.1, SD 16.6) in patients with XLH starting burosumab (33). Results from a further phase 3 extension trial by Briot et al. showed improvements at 24 weeks compared with baseline in WOMAC (least square mean -3.94, SD 2.19) and 6MWT (least square mean +2.56, SD 1.11) (27). Furthermore, a prospective case series in adolescent patients with XLH demonstrated an improvement in patient-reported outcomes of WOMAC and the revised Graded Chronic Pain Scale (GCPS-R) following 12 to 48 months of burosumab use (34). Additionally, serum phosphate, TmP/GFR and 1,25-dihydroxyvitamin D levels significantly increased following burosumab initiation while ALP levels significantly decreased (34). Critically, this case series demonstrated that these positive outcomes were not sustained following burosumab discontinuation, suggesting that on-going burosumab treatment throughout adolescence is essential (34). Early treatment with burosumab for symptomatic individuals aged 1 to 17 years with XLH is recommended in international guidelines (35). For symptomatic adults, treatment with oral phosphate and active vitamin D remains the standard of care, except for patients with pseudofractures or those who experience a lack of response or adverse events with oral phosphate and active vitamin D for whom burosumab treatment is recommended (35).

Although this study provides valuable insights into the impact of XLH in this cohort of 26 family members, there are some limitations to the study. The observational nature of the study and use of patient-reported outcomes can lead to some level of bias. Nevertheless, patient-reported outcomes are essential to understand quality of life in patients with XLH. Missing data for some patients is a further limitation; however, there were difficulties faced by some family members in attending appointments at KFSHRC due to the semi-remote location and poor socioeconomic status. While it is known that some the family members are from low socio-economic backgrounds, additional information on social engagement of the patients, level of instruction, and profession was not available. It would be useful to assess the impact of these factors on patient attendance at follow-up appointments as well as to understand how XLH impacts social engagement, education and work productivity. Future studies will aim to further assess HRQoL in patients with XLH in Saudi Arabia, especially in patients initiating burosumab treatment, to further understand how treatment can impact quality of life. Future work might also focus on the economic impact of the disease on the Saudi Arabia health system.

### 4.1 Conclusions

This study demonstrates the huge impact of XLH in 26 family members from Saudi Arabia who had delayed diagnosis and poor access to medical treatment and supportive care. Poor quality of life, skeletal deformities, and multiple complications and procedures are likely exacerbated by a lack of treatment in this set of patients. Greater awareness and early diagnosis of XLH are essential for identification of cases and early initiation of treatment. We propose that healthcare professionals should perform full family mapping for every patient diagnosed with XLH.

## Data Availability

The raw data supporting the conclusions of this article will be made available by the authors, without undue reservation.
